# Timing of First Acute Rejection and Long-Term Kidney Allograft Survival in the Contemporary Calcineurin-Inhibitor Era: A Single-Center Cohort Study (2000–2018)

**DOI:** 10.3390/jcm15145336

**Published:** 2026-07-08

**Authors:** Jungjun Lee, Sunyoung Son, Manki Ju

**Affiliations:** Department of Transplantation Surgery, Gangnam Severance Hospital, Yonsei University College of Medicine, 211 Eonju-ro, Gangnam-gu, Seoul 06273, Republic of Korea

**Keywords:** kidney transplantation, acute rejection, allograft survival, glomerular filtration rate, tacrolimus, cyclosporine, calcineurin inhibitor, risk stratification

## Abstract

**Background:** Acute rejection (AR) is an established risk factor for kidney allograft loss, but whether the timing of the first AR episode adds prognostic information independent of secular changes in immunosuppression remains incompletely defined. We re-examined this question in a cohort restricted to the contemporary calcineurin-inhibitor (CNI) era and explicitly accounted for the maintenance CNI (tacrolimus vs. cyclosporine). **Methods:** We studied 2470 recipients of an isolated kidney transplant performed between 2000 and 2018 at a single academic center. Recipients were classified by the timing of the first AR episode as AR-free, Early AR (≤6 months), Intermediate AR (6–12 months), or Late AR (>12 months). Co-primary outcomes were estimated glomerular filtration rate (eGFR) and death-censored graft survival. Multivariable Cox regression adjusted for recipient and donor age, recipient sex, HLA mismatch, donor type, ABO incompatibility, diabetes, retransplantation, transplant year, and maintenance CNI. A pre-specified secondary analysis evaluated whether the observed AR-timing association was explained by the maintenance CNI. **Results:** AR occurred in 294 of 2470 recipients (11.9%): 253 Early, 10 Intermediate, and 31 Late. Median follow-up was 105 months. Mean 5-year eGFR was 67.1 (AR-free), 56.5 (Early), 47.5 (Intermediate), and 47.2 mL/min/1.73 m^2^ (Late) (*p* < 0.001). Kaplan–Meier 10-year death-censored graft survival was 90.5%, 80.7%, 77.8%, and 52.2%, respectively (log-rank *p* < 0.001). After adjustment, the hazard of graft failure relative to AR-free recipients was 2.25 (95% CI 1.68–3.01) for Early AR and 7.03 (4.28–11.54) for Late AR (both *p* < 0.001). The Intermediate AR estimate was directionally consistent but imprecise because of the small number of cases. Tacrolimus use was associated with a lower observed incidence of AR (8.5% vs. 20.0%, *p* < 0.001), but the observational CNI comparison was confounded by era and follow-up duration and did not explain the AR-timing gradient. **Conclusions:** Within a contemporary CNI-era cohort and after adjustment for maintenance immunosuppression and transplant year, late-onset AR—particularly onset beyond the first post-transplant year—provided strong prognostic information for long-term graft dysfunction and graft failure.

## 1. Introduction

Kidney transplantation remains the optimal renal-replacement strategy for most patients with end-stage kidney disease, offering superior survival and quality of life compared with maintenance dialysis [1,2]. Despite substantial improvements in short-term outcomes, the long-term durability of the transplanted kidney has improved only modestly, and identifying the determinants of late allograft loss remains a central question in clinical transplantation [3,4].

Acute rejection (AR) is among the most consistently identified risk factors for graft failure across cohorts and eras [5,6,7]. The progressive adoption of calcineurin inhibitors, mycophenolate, and modern induction agents has reduced the incidence of early biopsy-proven rejection from approximately 40–50% in the cyclosporine era to 10–15% in contemporary tacrolimus-based regimens [8]. Yet the prognostic implications of an AR episode vary considerably with its histopathological character, severity, response to treatment, and—of particular relevance here—the interval between transplantation and its onset.

A growing body of evidence suggests that late AR, conventionally defined as rejection arising more than 6 to 12 months after transplantation, has a different natural history from early AR. Early AR predominantly reflects T-cell-mediated alloreactivity and usually responds to standard therapy, whereas late AR is enriched for antibody-mediated injury, chronic active rejection, and rejection driven by inadequate immunosuppressive exposure or non-adherence [9,10,11,12]. We adopted the 6- and 12-month cut-points because they retain clinical meaning in contemporary practice: the first 6 months represent the period of highest alloimmune activity and most intensive immunosuppression, when T-cell-mediated rejection predominates; the 6-to-12-month window is a transitional phase during which immunosuppression is commonly minimized; and rejection first arising beyond 12 months, once exposure has been reduced and the early high-risk period has passed, is disproportionately antibody-mediated or adherence-related and carries a distinct prognosis [9,10,11,12,13,14]. These thresholds also align with the intervals used in prior outcome studies, allowing comparison with the existing literature. A detailed account of the biological mechanisms underlying the late-rejection phenotype is deferred to the Discussion, where it can be considered alongside our findings rather than pre-empting them.

An important critique of long-horizon analyses of AR timing is that cohorts spanning several decades conflate the prognostic information carried by rejection timing with secular changes in immunosuppression, donor and recipient selection, surgical technique, and the histological definition of rejection itself. A multi-decade cohort that mixes cyclosporine/azathioprine-era and tacrolimus/mycophenolate-era recipients cannot easily separate the effect of AR timing from the era in which the transplant was performed. We therefore re-examined the question within a more homogeneous, contemporary cohort restricted to transplants performed from 2000 onward—after tacrolimus and mycophenolate had become widely used—and explicitly modeled the maintenance CNI (tacrolimus vs. cyclosporine) as an adjustment covariate. We also present a dedicated CNI comparison as a descriptive, non-causal analysis to evaluate whether the AR-timing association was merely a reflection of CNI-era differences.

We hypothesized that, even within the contemporary era and after accounting for the maintenance immunosuppressant, the timing of the first clinically significant AR episode—particularly onset beyond the first post-transplant year—would remain associated with worse long-term eGFR and graft survival.

## 2. Materials and Methods

### 2.1. Study Design, Population, and Ethics

We performed a single-center retrospective cohort study using the prospectively maintained kidney transplantation registry of Gangnam Severance Hospital, a tertiary academic transplant center in Seoul, Republic of Korea. To restrict the analysis to the contemporary immunosuppression era and to mitigate era heterogeneity, eligibility was limited to consecutive recipients of an isolated kidney transplant performed between January 2000 and December 2018; transplants performed before 2000, during the predominantly cyclosporine/azathioprine era, were excluded. The study complied with the Declaration of Helsinki and was approved by the Institutional Review Board of Gangnam Severance Hospital (IRB number 3-2019-0096).

### 2.2. Exposure Classification: Timing of Acute Rejection

AR was defined as a clinically diagnosed, biopsy-proven rejection episode treated with antirejection therapy and recorded in the registry, time-stamped at the date of diagnosis. Using the first documented episode, recipients were assigned to four mutually exclusive groups: AR-free (no documented AR during follow-up); Early AR (first episode ≤ 6 months after transplantation); Intermediate AR (first episode between 6 and 12 months); and Late AR (first episode > 12 months). The 6- and 12-month cut-points were pre-specified on the basis of prior literature distinguishing early T-cell-mediated rejection from later, more frequently antibody-mediated or adherence-related rejection [9,10,11,12]. Because the cohort, although confined to the contemporary era, still spans nearly two decades during which biopsy reporting standards and antibody-mediated rejection recognition evolved [15,16,17], AR was analyzed as a registry-defined, clinically treated, biopsy-proven event rather than by Banff category; consistent Banff subtyping, donor-specific antibody status, and treatment-response data were not available across the entire study period. Accordingly, AR timing should be interpreted primarily as a clinically useful prognostic marker rather than as proof of a distinct causal rejection phenotype. All rejection episodes were diagnosed on indication (for-cause) biopsies prompted by graft dysfunction; the program did not perform routine protocol (surveillance) biopsies during the study period, so subclinical rejection was not systematically ascertained. Borderline changes were not counted as AR unless the episode was clinically treated with antirejection therapy and recorded as a rejection event in the registry. Exposure was based on the first treated AR episode; recurrent or subsequent episodes after the first were not used for group assignment, and patients were classified once, by the timing of that first event. Where antibody-mediated, T-cell-mediated, or mixed phenotypes were documented, all were included under the single registry-defined AR category, because Banff subtype and donor-specific antibody status were not uniformly recorded across the cohort and could not support reliable phenotype-specific subgroup analysis.

### 2.3. Immunosuppression Classification

The initial maintenance calcineurin inhibitor recorded at discharge was classified as tacrolimus (Prograf, Tacrobell, or Advagraf) or cyclosporine (Sandimmun, Neoral, or Cipol-N). Three recipients without a recorded maintenance CNI were retained in the timing analyses but excluded from CNI-specific comparisons and from the adjusted model. The CNI variable was used both as an adjustment covariate in the multivariable model of AR timing and as the exposure in a pre-specified secondary comparison of tacrolimus- versus cyclosporine-treated recipients. Only the initial maintenance CNI recorded at discharge was analyzed. Subsequent within-patient changes in immunosuppression during follow-up—including CNI conversion (e.g., cyclosporine-to-tacrolimus switching), conversion to or from mTOR inhibitors or belatacept, dose adjustments, and measured CNI trough concentrations—as well as the duration of each regimen, were not captured in the registry and were therefore not modeled. The CNI variable should accordingly be read as the index maintenance agent rather than as a time-updated summary of cumulative immunosuppressive exposure.

### 2.4. Outcomes and Covariates

Co-primary outcomes were (i) eGFR at 1, 3, 5, and 10 years after transplantation, computed from registry serum creatinine using the CKD-EPI creatinine equation [18], and (ii) death-censored graft survival. Graft failure was defined as return to chronic dialysis, pre-emptive retransplantation, or graft nephrectomy; recipients who died with a functioning graft were censored at death for the death-censored analysis. Pre-specified secondary endpoints were overall graft loss (graft failure from any cause, including death with a functioning graft) and all-cause patient death. Covariates, selected a priori, were recipient age, donor age, recipient sex, total HLA-A/B/DR mismatch, donor type (living-related, living-unrelated, or deceased), ABO incompatibility, recipient pre-transplant diabetes, retransplantation, calendar year of transplantation, and maintenance CNI. All baseline covariates entered into the multivariable model, as well as the exposure (AR timing) and the death-censored graft-failure outcome, were complete for the analyzed cohort; the only missing values were the three recipients without a recorded maintenance CNI, who were retained in the timing analyses but excluded from the CNI-specific comparisons and from the adjusted Cox model (analyzed *n* = 2467). No imputation was performed. Serum creatinine values used to derive eGFR were not available at every landmark for every recipient because of variable visit timing and graft loss; eGFR at each landmark was therefore summarized among recipients with a functioning graft and an available creatinine value at that time point, and no eGFR values were imputed.

### 2.5. Statistical Analysis

Continuous variables were summarized as mean ± standard deviation and compared with the Kruskal–Wallis test; categorical variables were compared with the Pearson chi-squared test. eGFR at each landmark was compared across AR-timing groups by the Kruskal–Wallis test. Death-censored graft survival was estimated by the Kaplan–Meier method and compared by the multivariate log-rank test. Adjusted hazard ratios (HRs) with 95% confidence intervals were obtained from a multivariable Cox proportional hazards model including AR-timing group (reference: AR-free) and the covariates above; the proportional hazards assumption was assessed with scaled Schoenfeld residuals. Two pre-specified sensitivity analyses were performed: a landmark analysis among recipients whose grafts were functioning at 12 months (survival re-originated at the landmark) to address immortal time bias in the Late AR classification; and the tacrolimus-versus-cyclosporine comparison. Two-sided *p* < 0.05 was considered significant. Analyses used R 4.2.0 and Python 3.11 (pandas, lifelines, SciPy). Continuous variables are reported as mean ± standard deviation to aid comparison with prior transplant cohorts, but, because several distributions were skewed, between-group comparisons used the non-parametric Kruskal–Wallis test rather than parametric analysis of variance; medians with interquartile ranges gave concordant results. To address the concern that classifying acute rejection by the timing of the first episode treats a time-varying event as a fixed baseline exposure, and that the Late AR group is therefore subject to immortal time bias, we specified a time-dependent Cox proportional-hazards model as a co-primary survival analysis in addition to the fixed-classification model. In this model each recipient contributed AR-free person-time until the date of the first treated AR episode, after which an indicator for acute rejection (and, in a secondary parameterization, the corresponding timing category, ≤6, 6–12, or >12 months) was updated to reflect the post-rejection state, so that rejection status and timing entered the model as time-updated covariates with the same adjustment set as the fixed model. The proportional-hazards assumption was assessed for every covariate using scaled Schoenfeld residuals; the assumption was satisfied for the key Late AR term (*p* = 0.87) and for most covariates, with only the Early AR term showing a borderline signal (*p* = 0.02), which the landmark and time-dependent analyses were designed to address. The number of recipients with a functioning graft and an available serum creatinine value contributing to each landmark eGFR estimate is reported by group in Table 2 to make survivor bias at later landmarks transparent, and numbers at risk are displayed beneath the Kaplan–Meier curves (Figure 2 and Figure 4). As an additional pre-specified sensitivity analysis, the Intermediate and Late AR groups were combined into a single “rejection after 6 months” stratum to evaluate the robustness of the late-rejection association independent of the small Intermediate group. Because the registry contained no missing values for the exposure, the death-censored graft-failure outcome, or any covariate entered into the multivariable model (other than the three recipients without a recorded maintenance CNI, who were excluded from the adjusted model, analyzed *n* = 2467), no multiple imputation was required; eGFR landmark summaries used available creatinine values without imputation.

## 3. Results

### 3.1. Cohort Characteristics

A total of 2470 transplants performed between 2000 and 2018 met the eligibility criteria (1100 during 2000–2009 and 1370 during 2010–2018). Mean recipient age was 43.9 ± 12.5 years, mean donor age 41.1 ± 12.3 years, and 40.5% of recipients were female. Living related donors contributed 49.8% of grafts, living-unrelated donors 26.5%, and deceased donors 23.7%. Tacrolimus was the initial maintenance CNI in 1738 recipients (70.4%) and cyclosporine in 729 (29.5%). Median follow-up was 105 months (interquartile range 61–158).

AR was documented in 294 recipients (11.9%): 253 (10.2%) Early, 10 (0.4%) Intermediate, and 31 (1.3%) Late. Recipients in the AR groups were younger than AR-free recipients (*p* < 0.001), and tacrolimus use was higher in the AR-free group (73.1%) than in the rejection groups (50–52%, *p* < 0.001), consistent with the lower rejection rate observed with tacrolimus. Other baseline characteristics were broadly comparable (Table 1).

### 3.2. eGFR Trajectory by AR Timing

**Figure 1:**
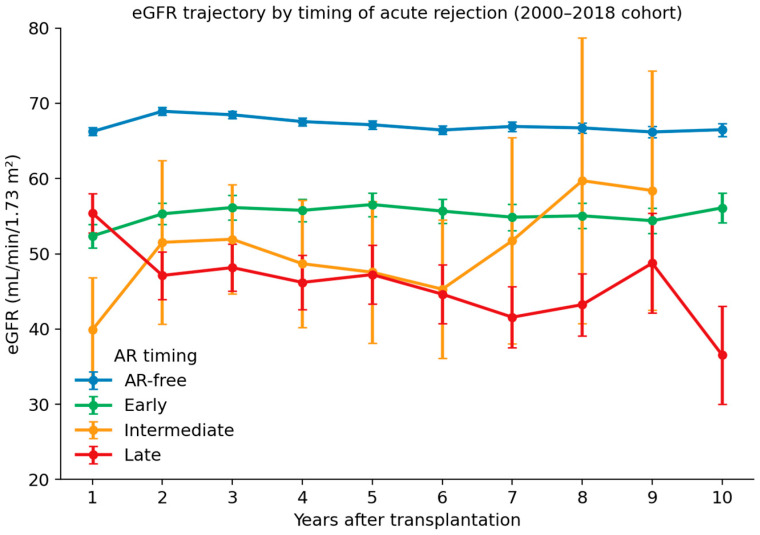
Long-term eGFR trajectory by timing of acute rejection in the 2000–2018 cohort. Symbols and error bars are group mean ± standard error at each annual landmark. The Late AR group starts closer to the AR-free level at 1 year and declines after late rejection emerges. The Intermediate group is small (*n* = 10), so its trajectory is descriptive rather than definitive. eGFR, estimated glomerular filtration rate; AR, acute rejection.

Mean eGFR diverged across AR-timing groups from the earliest landmark and the divergence persisted over time (Figure 1; Table 2). At 1 year, the Intermediate AR group already had a substantially lower mean eGFR (39.9 ± 21.9 mL/min/1.73 m^2^) than the AR-free group (66.2 ± 23.8), reflecting recent rejection injury, whereas the Late AR group—whose first rejection had not yet occurred by 1 year—started closer to the AR-free level (55.4 ± 14.5). This pattern reversed at later landmarks: Late AR eGFR fell to 48.2 ± 16.7 at 3 years, 47.2 ± 18.7 at 5 years, and 36.5 ± 21.6 at 10 years, while the AR-free group remained close to 66–67 mL/min/1.73 m^2^ throughout (Kruskal–Wallis *p* < 0.001 at every landmark). The Early AR group stabilized at an intermediate level near 56 mL/min/1.73 m^2^. Because Intermediate AR was uncommon (*n* = 10), its landmark estimates should be interpreted as descriptive and imprecise. The number of recipients with a functioning graft and an available creatinine value contributing to each landmark is now reported by group in Table 2. Because grafts are lost and follow-up shortens over time, the denominator at later landmarks is smaller—particularly in the small Intermediate and Late strata—so the 10-year means in these groups reflect survivors and should be read as descriptive; this survivor bias is one reason the cross-sectional landmark comparison cannot, on its own, characterize individual eGFR trajectories, and the limitations of this repeated-cross-section approach relative to a longitudinal mixed-effects or joint model are addressed in the Discussion.

### 3.3. Death-Censored Graft Survival

**Figure 2:**
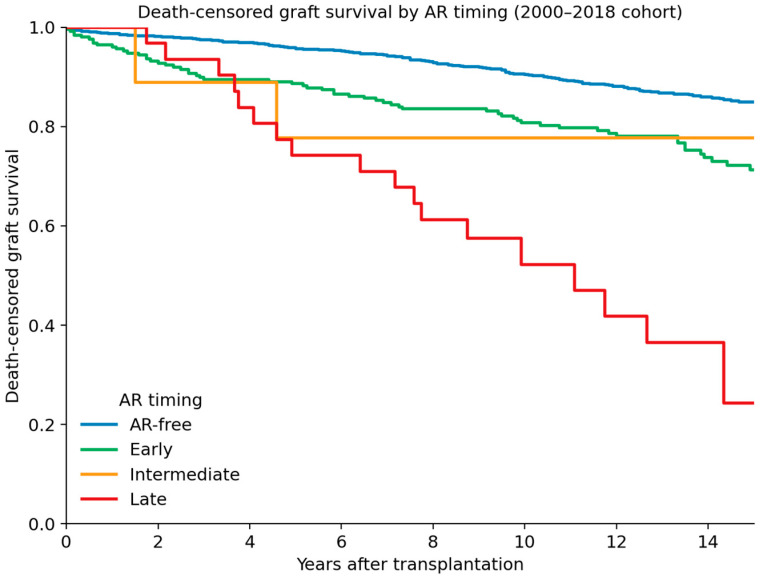
Kaplan–Meier estimates of death-censored graft survival by timing of first acute rejection (2000–2018 cohort). The Late AR curve separates after the first year and falls to roughly 52% at 10 years, versus 81% for Early AR and 91% for AR-free recipients. AR, acute rejection.

Death-censored graft survival diverged markedly across AR-timing groups (Figure 2). Kaplan–Meier estimates of survival at 3, 5, and 10 years were 97.5%, 95.7%, and 90.5% for AR-free; 89.5%, 88.6%, and 80.7% for Early AR; 88.9%, 77.8%, and 77.8% for Intermediate AR; and 93.5%, 74.2%, and 52.2% for Late AR. The multivariate log-rank test was highly significant (χ^2^ = 95.8, 3 df, *p* < 0.001). The Late AR curve initially tracked the AR-free curve and then descended steeply after the first post-transplant year, ultimately reaching the lowest 10-year survival. The Intermediate AR curve should be interpreted cautiously because only 10 recipients contributed to this stratum.

### 3.4. Adjusted Hazards

**Figure 3:**
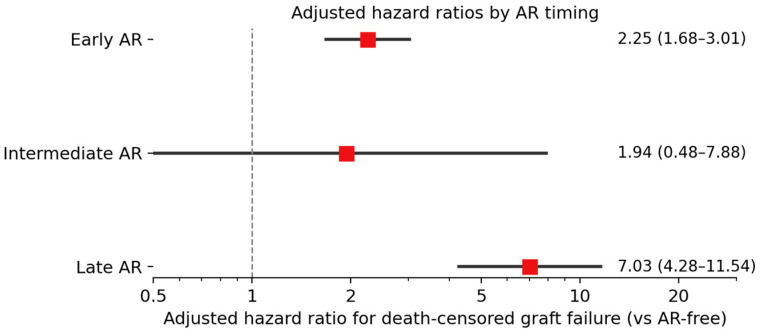
Forest plot of adjusted hazard ratios (95% CI) for death-censored graft failure by AR timing, with AR-free recipients as reference, from the multivariable Cox model in Table 3. The estimates are displayed on a logarithmic *x*-axis because the Late AR estimate has a wide confidence interval. AR, acute rejection; HR, hazard ratio; CI, confidence interval. Dashed line indicates the AR-free reference group. AR-, AR-free (no acute rejection).

In the multivariable Cox model (*n* = 2467; 288 events; concordance index 0.68) adjusting for recipient and donor age, recipient sex, HLA mismatch, donor type, ABO incompatibility, diabetes, retransplantation, transplant year, and maintenance CNI, the adjusted HR for death-censored graft failure relative to AR-free recipients was 2.25 (95% CI 1.68–3.01, *p* < 0.001) for Early AR and 7.03 (4.28–11.54, *p* < 0.001) for Late AR (Figure 3; Table 3). The Intermediate AR estimate was directionally consistent but imprecise owing to the small number of such recipients (HR 1.94, 0.48–7.88, *p* = 0.36). Because only 10 recipients contributed to the Intermediate AR stratum, this hazard ratio is unstable, and its wide confidence interval includes both no effect and a large effect; it should therefore be regarded as descriptive and hypothesis-generating only, and should not be over-interpreted or used for clinical risk stratification. Other independent predictors of graft failure were older donor age, increasing HLA mismatch, deceased-donor source, and diabetes. Maintenance CNI was not independently associated with death-censored graft failure (tacrolimus vs. cyclosporine HR 1.03, 0.78–1.36, *p* = 0.85), indicating that the strong association between Late AR and graft failure was not explained by the recorded choice of calcineurin inhibitor.

### 3.5. Tacrolimus Versus Cyclosporine

**Figure 4:**
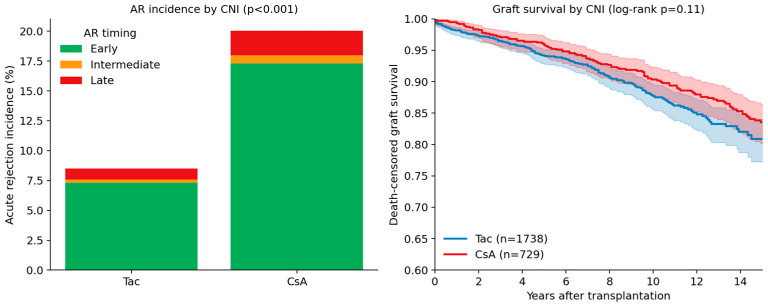
Tacrolimus versus cyclosporine. (**Left**): stacked acute rejection incidence by CNI (*p* < 0.001). (**Right**): Kaplan–Meier death-censored graft survival by CNI with 95% confidence intervals (log-rank *p* = 0.11). This descriptive comparison is confounded by calendar era and follow-up duration. CNI, calcineurin inhibitor. The blue and red shaded areas represent the 95% confidence intervals for the respective groups.

In the pre-specified descriptive comparison of maintenance CNI, tacrolimus-treated recipients (*n* = 1738) had a markedly lower observed incidence of AR than cyclosporine-treated recipients (*n* = 729): any AR 8.5% vs. 20.0% and early AR 7.3% vs. 17.3% (*p* < 0.001; Figure 4, Table 4). Mean 5-year eGFR was modestly higher with tacrolimus (66.6 vs. 63.9 mL/min/1.73 m^2^; *p* = 0.011). Death-censored graft survival did not differ significantly between groups (10-year survival 87.7% with tacrolimus vs. 90.3% with cyclosporine; log-rank *p* = 0.11), but this comparison is strongly influenced by era and follow-up duration (median 86 vs. 163 months), reflecting the earlier predominance of cyclosporine within the 2000–2018 window. Therefore, the CNI comparison should be interpreted as supportive and descriptive rather than as a causal estimate of the effect of tacrolimus on graft survival.

### 3.6. Secondary Endpoints

Overall graft loss rose monotonically with later rejection, from 14.8% (AR-free) to 37.5% (Early), 40.0% (Intermediate), and 58.1% (Late) (chi-squared *p* < 0.001; Table 5). All-cause patient death did not follow the same gradient (6.2%, 11.1%, 20.0%, and 0.0%, respectively; *p* = 0.003), indicating that the excess overall graft loss in the late-rejection strata was driven by death-censored graft failure rather than by mortality, consistent with the primary analysis.

### 3.7. Sensitivity Analysis

In the landmark analysis restricted to recipients whose grafts were functioning at 12 months, with follow-up re-originated at that landmark, Late AR remained strongly associated with death-censored graft failure (adjusted HR 7.57, 95% CI 4.59–12.48; *p* < 0.001), as did Early AR (HR 2.12, 1.55–2.89; *p* < 0.001); the Intermediate estimate was again imprecise (HR 2.07, 0.51–8.44; *p* = 0.31). The persistence of the Late AR association after re-origination indicates that its adverse prognosis is unlikely to be explained solely by immortal time bias. In the co-primary time-dependent Cox model, in which acute rejection and its timing category were modeled as time-updated exposures (each recipient contributing AR-free person-time until the first treated episode), the association between rejection and death-censored graft failure was consistent with the fixed-classification analysis: relative to AR-free person-time, the adjusted hazard of graft failure was 2.15 (95% CI 1.60–2.90) for rejection occurring at ≤6 months and 8.55 (5.19–14.08) for rejection occurring at >12 months (both *p* < 0.001), with the >12-month stratum again carrying the highest hazard; the 6–12-month estimate remained imprecise (HR 2.18, 0.54–8.88, *p* = 0.28). Modeling rejection as a time-updated exposure therefore did not attenuate the late-rejection gradient—and, if anything, strengthened it—indicating that the association is not an artifact of immortal time in the fixed classification. In the additional sensitivity analysis combining the Intermediate and Late groups into a single “rejection after 6 months” stratum (*n* = 41), this combined late-onset group remained strongly associated with death-censored graft failure (adjusted HR 5.49, 95% CI 3.42–8.80; *p* < 0.001), confirming that the late-rejection signal does not depend on the small Intermediate stratum.

## 4. Discussion

In a contemporary single-center cohort of 2470 kidney transplant recipients transplanted between 2000 and 2018 and followed for a median of 105 months, the timing of the first clinically significant AR episode provided independent prognostic information for long-term eGFR and death-censored graft survival. The most clinically important finding was the poor prognosis of late-onset rejection: AR first emerging beyond the first post-transplant year carried an adjusted hazard of graft failure approximately three-fold higher than early rejection and seven-fold higher than that of recipients who never rejected. These findings held after restricting the cohort to the contemporary calcineurin-inhibitor era and after adjusting for maintenance immunosuppression and transplant year, addressing the concern that the prognostic information carried by AR timing might be an artifact of secular changes in immunosuppression.

### 4.1. Addressing Era and Immunosuppression Heterogeneity

A central methodological concern with long-horizon analyses of AR timing is that a study window spanning several decades conflates the biology of rejection timing with era effects. We addressed this in two ways. First, by confining the cohort to 2000–2018, we removed the cyclosporine/azathioprine-dominant period and analyzed a population managed under broadly modern protocols. Second, by classifying every recipient by maintenance CNI and entering it into the model, we directly controlled for the single most influential immunosuppressive change of the era—the shift from cyclosporine to tacrolimus. The AR-timing gradient persisted in this more homogeneous cohort, and the CNI itself was not independently associated with graft survival (HR 1.03), arguing that the timing effect reflects the biology of when rejection occurs rather than the era in which it was treated. This interpretation should nonetheless be tempered. Restricting the cohort to 2000–2018 and adjusting for the maintenance CNI does not render the period immunologically homogeneous: induction therapy, tacrolimus target levels, mycophenolate use, donor-specific antibody monitoring, biopsy indications, evolving Banff criteria, recognition of antibody-mediated rejection, and supportive care all changed materially across these two decades. The initial maintenance CNI captures only one dimension of contemporary immunosuppressive exposure and is not a complete proxy for it. We therefore frame the cohort as more homogeneous than a multi-decade cyclosporine-to-tacrolimus span, not as era-homogeneous, and we cannot exclude residual confounding by these unmeasured, time-varying era factors; the persistence of the AR-timing gradient is consistent with, but does not prove, a predominantly biological explanation.

The dedicated tacrolimus-versus-cyclosporine comparison is informative but must be interpreted cautiously. Tacrolimus use was associated with a substantially lower observed incidence of AR (8.5% vs. 20.0%) and modestly higher 5-year eGFR, consistent with randomized and registry evidence that tacrolimus reduces acute rejection [8]. However, the comparison of long-term graft survival between CNI groups is confounded by calendar era, differing follow-up duration, and potential differences in donor and recipient selection. Accordingly, these data should not be read as showing that tacrolimus has no causal effect on graft survival; rather, they indicate that the poor prognosis associated with late-onset AR was not explained by the recorded maintenance CNI in this cohort.

### 4.2. Comparison with Prior Literature

Our results extend a literature that has progressively established late AR as a marker of accelerated allograft loss [19,20]. Sijpkens and colleagues found that rejection after 3 months carried worse graft survival than earlier episodes [11], and Joseph et al. reported 10-year graft survival of approximately 28% for late AR [21]. In a single-center Korean cohort from the tacrolimus era, Koo and colleagues reported time-dependent adjusted hazard ratios of 3.37 for early and 5.32 for late AR—point estimates close to ours and supporting external validity in a comparable population [22]. In the multicenter Collaborative Transplant Study, Opelz and Döhler likewise showed that later rejection conferred a progressively greater hazard of subsequent graft loss [23]. Mechanistically, Sellàres and colleagues found that antibody-mediated rejection and non-adherence together accounted for the majority of late graft failures [13], while Wiebe and coworkers showed that de novo donor-specific antibody typically precedes clinical late rejection and predicts graft loss [14]; Gaston and colleagues similarly identified antibody-mediated injury as the dominant driver of late failure [24,25]. These lines of evidence support a biological, rather than purely confounding, explanation for the prognostic gap between early and late rejection.

### 4.3. Mechanisms Underlying the Late-AR Phenotype

Three non-mutually-exclusive mechanisms may explain why late AR is a high-risk clinical phenotype. First, late clinically significant rejection may be enriched for antibody-mediated rejection and transplant glomerulopathy, lesions comparatively refractory to current therapy; even histologically T-cell-mediated late episodes, when accompanied by inflammation in areas of fibrosis and atrophy (i-IFTA), predict graft failure [26,27,28,29,30,31,32]. Second, waning adherence to immunosuppression over time can contribute to late rejection and poor response to conventional rescue therapy [33,34]. Third, by the time late rejection occurs, the allograft may have accumulated subclinical injury, so an acute immune insult superimposed on already-compromised parenchyma can produce a disproportionate and frequently irrecoverable functional decrement [3,5,6,35]. Because Banff subtype, donor-specific antibody status, drug-level variability, adherence, and treatment response were not uniformly captured, our data should be interpreted as showing that late AR captures adverse prognostic information, not as proving a single causal biological pathway.

### 4.4. Clinical Implications

Several practical inferences follow. Although late AR was uncommon in this contemporary cohort (1.3%), its consequences were severe, with 10-year death-censored graft survival near 52%. Programs that concentrate surveillance in the first year and de-escalate sharply thereafter may miss the rejection events that matter most for long-term survival. Where resources permit, de novo donor-specific antibody monitoring beyond the first year is a rational extension of standard care, and structured adherence support—particularly during life transitions—should be considered standard rather than reactive. Finally, an uneventful first year should not be taken to exempt a recipient from later immunological hazard: the patient who tolerates the first year but then develops late AR faces a prognosis at least as poor as one who experienced and recovered from early rejection.

### 4.5. Strengths and Limitations

Strengths include the contemporary, era-restricted design, the explicit modeling of the maintenance CNI, the long follow-up (median 105 months), death-censoring of competing mortality, and the uniformity of diagnosis and management afforded by a single center. Several limitations warrant acknowledgment. First, as a single-center study with a living-donor-predominant case mix, generalization to deceased-donor-dominant programs should be cautious. Second, AR was registry-defined from clinically diagnosed and treated episodes; without routine protocol biopsies, subclinical rejection—especially subclinical antibody-mediated rejection—was under-ascertained, so the true incidence of late immune activation is likely higher than the 1.3% we report, and consistent Banff subtyping was not available across the study period. The analysis therefore characterizes the timing, not the histological type, of clinically significant rejection. Third, the Intermediate AR group was small (*n* = 10), and its estimates are imprecise; the principal clinically interpretable contrast is between Early and Late AR. Fourth, the Late AR classification is subject to immortal time bias, which would tend to make late rejection appear better, not worse; the landmark analysis indicates that the Late AR association is unlikely to be solely an artifact of this bias. Fifth, although the analysis was restricted to the post-2000 CNI era and adjusted for calendar year and maintenance CNI, residual heterogeneity related to evolving induction therapy, DSA monitoring, biopsy practice, AMR recognition, tacrolimus target levels, and supportive care across the 2000–2018 period cannot be fully excluded. Sixth, the comparison of tacrolimus and cyclosporine is observational and confounded by indication and by differing follow-up duration, and should not be read as a causal estimate of CNI effect on graft survival. Seventh, several variables of established prognostic relevance were not captured uniformly across the two-decade study period and could not be incorporated as covariates, leaving the potential for residual confounding. In particular, consistent Banff subtyping (including the distinction of T-cell-mediated from antibody-mediated rejection and the grading of inflammation in areas of fibrosis and tubular atrophy), donor-specific antibody status, and structured medication-adherence data were not available for the full cohort. Because late AR is enriched for antibody-mediated injury and non-adherence, the prognostic information attributed to AR timing may partly reflect unmeasured differences in rejection phenotype, alloantibody, and adherence rather than timing alone; the timing effect should accordingly be interpreted as a clinically useful prognostic marker rather than as evidence of an isolated causal mechanism. Finally, induction regimen, CNI trough variability, medication adherence, treatment response after rejection, BK polyomavirus, and de novo donor-specific antibody were not captured with sufficient granularity for inclusion in the model. Three further methodological limitations deserve emphasis. First, although we now report a time-dependent Cox model alongside the fixed-classification and landmark analyses, all three approaches share the same registry-defined exposure, and no design can fully eliminate the possibility that unmeasured factors distinguishing recipients who reject late from those who never reject contribute to the observed gradient. Second, the death-censored graft-failure model showed only moderate discrimination (concordance index 0.68); AR timing is a clinically useful prognostic marker but, by itself, is far from a complete predictor of long-term graft loss, and the model is intended for inference on the AR-timing association rather than as a validated individual-risk prediction tool. Third, the eGFR analysis is based on cross-sectional means at fixed landmarks compared with non-parametric tests; this repeated-cross-section approach does not account for within-patient correlation, informative missingness, or graft loss before later landmarks, and is therefore vulnerable to survivor bias at 5 and 10 years. A longitudinal mixed-effects model or a joint model of eGFR and graft failure would characterize individual trajectories more rigorously, and our eGFR findings should be regarded as descriptive of group-level differences rather than as definitive trajectory estimates.

## 5. Conclusions

Within a contemporary, calcineurin-inhibitor-era cohort, and after adjustment for maintenance immunosuppression and transplant year, late-onset acute rejection was associated with, and provided strong prognostic information for, long-term eGFR decline and death-censored graft failure. Late acute rejection was associated with a 10-year death-censored graft survival of approximately 52%, compared with 81% for early rejection and 91% for recipients who never rejected (adjusted HR 7.03 and 2.25, respectively). These are observational associations: the data indicate that late acute rejection is a strong marker of adverse long-term outcomes, but do not establish that it is a proven cause of graft loss, and the absence of histological, donor-specific antibody, and adherence data means residual confounding cannot be excluded. The Intermediate AR estimate was imprecise because of the small number of cases. Tacrolimus use was associated with a lower observed incidence of rejection, but the adverse prognosis of late-onset AR was not explained by the recorded maintenance CNI. These findings support sustained immunological surveillance and adherence support throughout the post-transplant course and argue that risk-stratification frameworks should incorporate the timing—not merely the occurrence—of clinically significant rejection.

## Figures and Tables

**Table 1:** Baseline characteristics by timing of first acute rejection.

Characteristic	AR-Free (*n* = 2176)	Early (*n* = 253)	Intermediate (*n* = 10)	Late (*n* = 31)	*p*
Recipient age, y	44.3 ± 12.5	41.9 ± 11.4	38.2 ± 15.0	36.3 ± 12.3	<0.001
Donor age, y	41.1 ± 12.4	41.1 ± 12.3	37.6 ± 12.1	41.3 ± 11.3	0.84
Female recipient, %	41.0	38.7	30.0	22.6	0.16
HLA-A/B/DR mismatch	2.8 ± 1.5	2.9 ± 1.2	3.0 ± 1.6	2.7 ± 1.2	0.96
Deceased donor, %	24.3	20.6	20.0	6.5	0.07
ABO-incompatible, %	28.5	23.7	10.0	19.4	0.15
Diabetes, %	23.2	30.0	10.0	25.8	0.07
Retransplant, %	9.3	10.7	0.0	6.5	0.60
Tacrolimus, %	73.1	50.2	50.0	51.6	<0.001

Values are mean ± SD or percentage. *p* values: Kruskal–Wallis (continuous) or chi-squared (categorical). AR, acute rejection; HLA, human leukocyte antigen; SD, standard deviation. Except for recipient age and maintenance CNI (both *p* < 0.001), the apparent between-group differences in baseline characteristics did not reach statistical significance (all *p* ≥ 0.05); numerical differences across the AR-timing groups, including those in the small Intermediate and Late strata, should therefore be interpreted with caution and not as established disparities.

**Table 2:** Estimated glomerular filtration rate (mL/min/1.73 m^2^) by AR-timing group and post-transplant interval.

Interval	AR-Free, Mean ± SD	Early, Mean ± SD	Intermediate, Mean ± SD	Late, Mean ± SD
1 year	66.2 ± 23.8	52.4 ± 24.1	39.9 ± 21.9	55.4 ± 14.5
3 years	68.5 ± 22.3	56.2 ± 24.0	51.9 ± 19.2	48.2 ± 16.7
5 years	67.1 ± 22.0	56.5 ± 22.5	47.5 ± 23.1	47.2 ± 18.7
10 years	66.5 ± 23.9	56.1 ± 24.3	46.2 ± 19.9	36.5 ± 21.6

Values are mean ± SD. Kruskal–Wallis *p* < 0.001 at every landmark. The Intermediate group is small (*n* = 10), and its estimates are imprecise; therefore, the principal clinically interpretable contrast is between Early and Late AR. The number of recipients with a functioning graft and an available creatinine value contributing to each landmark (n at 1/3/5/10 years) was 2115/1901/1554/784 for AR-free, 238/217/206/153 for Early, 10/7/6/2 for Intermediate, and 31/29/23/11 for Late; later landmarks have progressively smaller denominators, so the corresponding means represent survivors and are descriptive.

**Table 3:** Multivariable Cox model for death-censored graft failure (*n* = 2467; 288 events).

Covariate	Adjusted HR (95% CI)	*p*
Early AR (vs. AR-free)	2.25 (1.68–3.01)	<0.001
Intermediate AR (vs. AR-free)	1.94 (0.48–7.88)	0.36
Late AR (vs. AR-free)	7.03 (4.28–11.54)	<0.001
Recipient age, per year	0.99 (0.98–1.00)	0.007
Donor age, per year	1.02 (1.01–1.03)	0.001
Female recipient	0.99 (0.78–1.26)	0.96
HLA mismatch, per locus	1.15 (1.05–1.27)	0.003
Deceased donor (vs. living-related)	1.50 (1.08–2.09)	0.015
Living-unrelated (vs. living-related)	0.82 (0.60–1.12)	0.21
ABO-incompatible	1.04 (0.78–1.39)	0.80
Diabetes	1.49 (1.13–1.96)	0.004
Retransplant	1.03 (0.69–1.54)	0.88
Tacrolimus (vs. cyclosporine)	1.03 (0.78–1.36)	0.85
Transplant year, per year	1.03 (0.99–1.07)	0.12

HR, hazard ratio; CI, confidence interval; AR, acute rejection; HLA, human leukocyte antigen. Concordance index = 0.68.

**Table 4:** Comparison of tacrolimus- and cyclosporine-treated recipients.

Variable	Tacrolimus (*n* = 1738)	Cyclosporine (*n* = 729)
Recipient age, year	44.8	41.9
Deceased donor, %	27.8	13.4
ABO-incompatible, %	30.7	21.0
Any acute rejection, %	8.5	20.0
Early acute rejection, %	7.3	17.3
Mean 5-year eGFR	66.6	63.9
10-year DC graft survival, %	87.7	90.3
Median follow-up, months	86	163

Any-AR difference *p* < 0.001; 5-year eGFR *p* = 0.011 (Mann–Whitney); death-censored (DC) graft survival log-rank *p* = 0.11. eGFR in mL/min/1.73 m^2^. AR, acute rejection.

**Table 5:** Overall graft loss, death-censored graft loss, and patient death by AR timing.

Endpoint	AR-Free	Early	Intermediate	Late
Overall graft loss, *n*/*N* (%)	322/2176 (14.8)	95/253 (37.5)	4/10 (40.0)	18/31 (58.1)
Death-censored graft loss, *n*/*N* (%)	200/2176 (9.2)	69/253 (27.3)	2/10 (20.0)	18/31 (58.1)
All-cause patient death, *n*/*N* (%)	135/2176 (6.2)	28/253 (11.1)	2/10 (20.0)	0/31 (0.0)

Chi-squared *p* < 0.001 for overall graft loss and *p* = 0.003 for patient death. Counts are shown as *n*/*N* (%) to make the small Intermediate and Late AR strata transparent. AR, acute rejection.

## Data Availability

The de-identified data underlying the analyses are available from the corresponding author upon reasonable request. The data are not publicly available owing to privacy and ethical restrictions.
